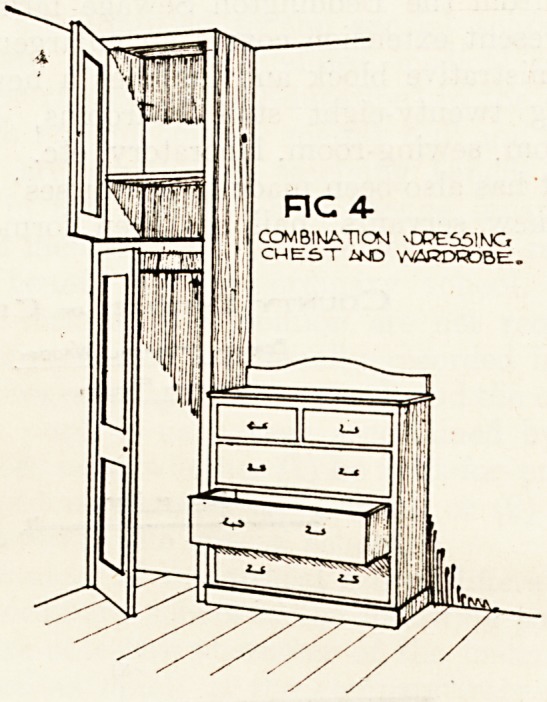# Borough Hospital, Waddon

**Published:** 1912-01-06

**Authors:** 


### HOSPITAL ARCHITECTURE AND CONSTRUCTION.

[Communications on this subject should be marked "Architecture" in the left-hand top corner of the envelope.]

### Borough Hospital, Waddon.

As reported in our issue of October 28, 1911, the above hospital extensions were opened on October 23, after an interesting résumé of progress by Sir Frederick Edridge. The original hospital site was some eight acres in extent in 1891, and to allow for extension three acres have been acquired recently from the Beddington Sewage farm.

The present extension covers an enlargement of the administrative block and includes a new wing containing twenty-eight staff bedrooms, nurses' sitting-room, sewing-room, laboratory, etc. An enlargement has also been made to the nurses' dining-room, a new servants' hall has been formed and charge nurse can see easily each bed under her supervision, the divisions being of plate glass. A careful study has been made of the ventilation problem, and the sketch, fig. 2, illustrates the system.

Each pavilion is surrounded by an open verandah and each cubicle can only be entered from its own outside door. Lavatory basins with elbow handle and blouse cupboards are placed in the wall on the verandah side.

Heating mains are taken longitudinally down the centre of the pavilion and radiators are placed in each apartment. The walls are built with an air

Section A B
FIG 2

Ground Plan
FIG I

Geo. F. Carter M.Inst.C.E.

Borough Engineer

October 1911

also waiting-room and offices. The new administrative wing is three storeys in height and approached on the several floors from the original building. The original staircase, however, being rather narrow an extra external iron staircase has been provided for exit in cases of panic and iron doors have been fitted on each floor to prevent the spread of fire.

Other sundry alterations about the original buildings include improvements in the disinfecting apparatus, new laundry sorting-room, additional coach-house, stable and workshop.

Undoubtedly the most interesting feature of the extension is the two observation isolation pavilions. Each contains twelve cubicle wards. A reference to our illustration, fig. 1, will show the general arrangement, which is on the lines suggested by the medical officer and put into working form by the borough engineer. It will be seen that the space to ensure an equable temperature in the cubicles.

All the new buildings are lighted by electricity and the Committee purpose to light all the old buildings in the same way, extending the cost over a few years.

It is interesting to note the gradual growth of this hospital from its inception:—

| | | | |
|---|---|---|---|
| First opened in 1893 | for | 40 | patients |
| Extended in 1896 | making total | 84 | " |
| Extended in 1900 | " | 146 | " |
| Extended in 1911 | " | 170 | " |

The building is of fire-resisting construction and the floors are finished with stout linoleum fixed to the cement concrete floor.

The nurses' bedrooms are about 12 feet by 8 feet, and 10 feet high. They comply with a modern standard in so far that no more floor space is occupied by furniture than is absolutely necessary. Reference to fig. 3 will show the arrangement. An

FIG 3
TYPICAL PLAN OF NURSE'S BEDROOM

iron lavatory basin is fitted in the corner beside the window, with hot and cold water laid on, and the unsightly but necessary pipes are enclosed in a small cupboard. Each bedroom has its fireplace for ventilation if not for use and over each is fixed a mirror about 24×18. Fig. 4 illustrates the combination dressing-chest and wardrobe. It is made of well-seasoned pine twice stained and twice varnished.

The wardrobe is divided into two compartments and the upper part is used for hats. The chest of drawers is of 1-inch grooved and tongued

FIG 4
COMBINATION DRESSING CHEST AND WARDROBE.

sides, the drawers have $\frac{3}{4}$-inch sides and bottoms and 1-inch front, with dovetailed joints and run on oak runners.

The cost of the new works, which have extended over some fourteen months, is about £11,000, and this sum includes the furnishings. We are indebted to Croydon's Borough Engineer, Mr. Geo. F. Carter, M.Inst.C.E., for permission to illustrate these works.

## Figures and Tables

**Fig 2 Fig 1 f1:**
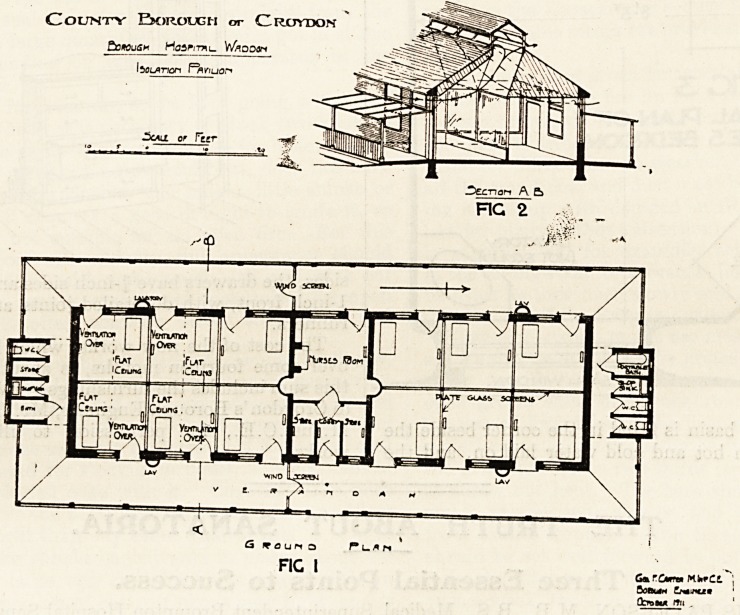


**Fig 3 f2:**
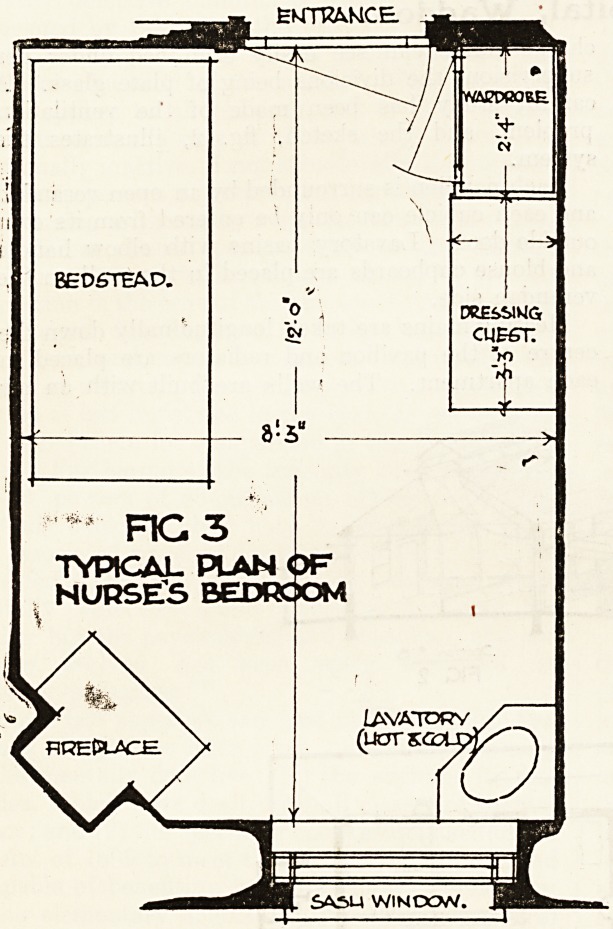


**Fig 4 f3:**